# Managing Childhood and Adolescent Atopic Dermatitis in Primary Care: A US Expert Group Consensus

**DOI:** 10.1016/j.jpedcp.2024.200121

**Published:** 2024-07-10

**Authors:** Mark Boguniewicz, Moise L. Levy, Lawrence F. Eichenfield, Christine T. Lauren, Donald Y.M. Leung, Lynda C. Schneider, Elaine C. Siegfried, Wynnis L. Tom, Amy S. Paller

**Affiliations:** 1 Division of Allergy-Immunology, Department of Pediatrics, National Jewish Health, Denver, CO; 2 University of Colorado School of Medicine, Denver, CO; 3 Department of Pediatrics and the Department of Internal Medicine (Dermatology), Dell Medical School at The University of Texas at Austin, Austin, TX; 4 Dell Children's Medical Center, Austin, TX; 5 Departments of Dermatology and Pediatrics, University of California San Diego, San Diego, CA; 6 Division of Pediatric and Adolescent Dermatology, Rady Children's Hospital, San Diego, CA; 7 Departments of Dermatology and Pediatrics, Columbia University Vagelos College of Physicians and Surgeons, New York, NY; 8 Division of Immunology, Boston Children's Hospital, Boston, MA; 9 Department of Pediatrics, Saint Louis University, St Louis, MO; 10 Department of Pediatric Dermatology, Cardinal Glennon Children's Hospital, St Louis, MO; 11 Departments of Dermatology and Pediatrics, Northwestern University Feinberg School of Medicine, Chicago, IL

**Keywords:** pediatric eczema, topical therapy, community care, primary care providers, systemic therapy

## Abstract

**Objective:**

This expert-led consensus aims to provide primary care providers (PCPs) with recommendations for the care of atopic dermatitis (AD) in patients aged <18 years. The first point of contact for diagnosis and management of AD is often a PCP, and appropriate, coordinated care between PCPs and AD specialists is essential to optimizing care.

**Study design:**

A systematic literature review was conducted followed by expert-led development of 25 consensus management recommendations relevant to 4 key themes in AD management: defining control, current and emerging treatments, referral care pathways, and patient-caregiver experience. Consensus was achieved using a modified Delphi process. For each statement, consensus for inclusion was considered achieved if ≥75% of the experts voted within the 7-9 range on a 9-point scale.

**Results:**

Consensus was reached on 24 of 25 statements. Nine statements reached the score of 7-9 by 100% of the experts. Of these, 4 were pertinent to topical therapy for the management of childhood and adolescent AD in primary care: the need for anti-inflammatory medication to achieve clear or almost clear skin; the need to tailor decisions about therapy to the individual patient or family; the importance of coordinated management between PCPs and specialists as part of effective treatment approaches; and the importance of patient and/or caregiver engagement in shared decision-making.

**Conclusions:**

It is hoped that these recommendations will guide the management of pediatric AD in primary care settings, facilitate coordinated care between PCPs and AD specialists, and improve outcomes for patients and their families.

Atopic dermatitis (AD) is a common, chronic, relapsing, inflammatory skin disease affecting 15% of children aged <16 years and characterized by intense pruritus, disruption of skin barrier function, and immune dysregulation.^1^ AD most often presents during infancy, with ≤90% of affected individuals developing symptoms before the age of 5.^2^^,^^3^ Patients frequently have atopic (asthma, allergic rhinitis, food allergy, and eosinophilic esophagitis) and nonatopic (especially infections) comorbidities.^4^, ^5^, ^6^ In addition, children with AD often have mental health comorbidities, including sleep disturbance, depression, anxiety, and attention deficit hyperactivity disorder.^7^^,^^8^ Further, time spent on skincare and missed workdays are a significant burden for caregivers, which increases with disease severity.^9^ It is clear that AD can have a substantial toll on the quality of life (QoL) of patients and their caregivers.^10^

The first healthcare point of contact for many children with AD is a primary care provider (PCP) and approximately one-half of patients with AD are treated exclusively in the primary care setting.^11^ In addition to treating patients with mild-to-moderate disease, PCPs are pivotal in referring children with moderate-to-severe disease for specialist care and assisting with maintenance care after specialty consultation (eg, managing acute infections, helping with systemic drug administration and/or monitoring).^12^ This expert-led consensus aims to provide PCPs with core recommendations for AD management in patients aged <18 years.

## Methods

In January 2022, a US-based group of 6 dermatologists and 3 allergists, selected based on their experience and expertise in AD, convened online to identify key questions concerning the management of AD in children and adolescents in a primary care setting.

A systematic literature review was conducted using Medline and EMBASE to identify publications relevant to 4 key themes relating to AD management: defining control, current and emerging treatments, referral care pathways, and the patient-caregiver experience. Search strings incorporated terms aligned to each of these themes. Initial search results were then filtered for relevance by title and abstract. Full details of the literature search can be found in the supplement. The expert panel then reviewed the literature results and developed 25 management recommendation statements based on the initial questions in facilitated virtual/email discussions. Draft statements were reviewed and refined independently by the panel in November 2022, and submitted to 2 rounds of voting using an online platform in January and February 2023. Consensus was achieved via modified Delphi methodology. During each round of voting, individual members voted anonymously on each statement using a 9-point scale (1 = strong disagreement; 9 = strong agreement). For each statement, consensus for inclusion was considered achieved if ≥75% of the experts voted within the 7-9 range on the 9-point scale, or for exclusion if ≥75% of the experts voted within the 1-3 range. A second round of voting and refinement was needed for statements that did not achieve the threshold for consensus or exclusion.

## Results

**Figure 1:**
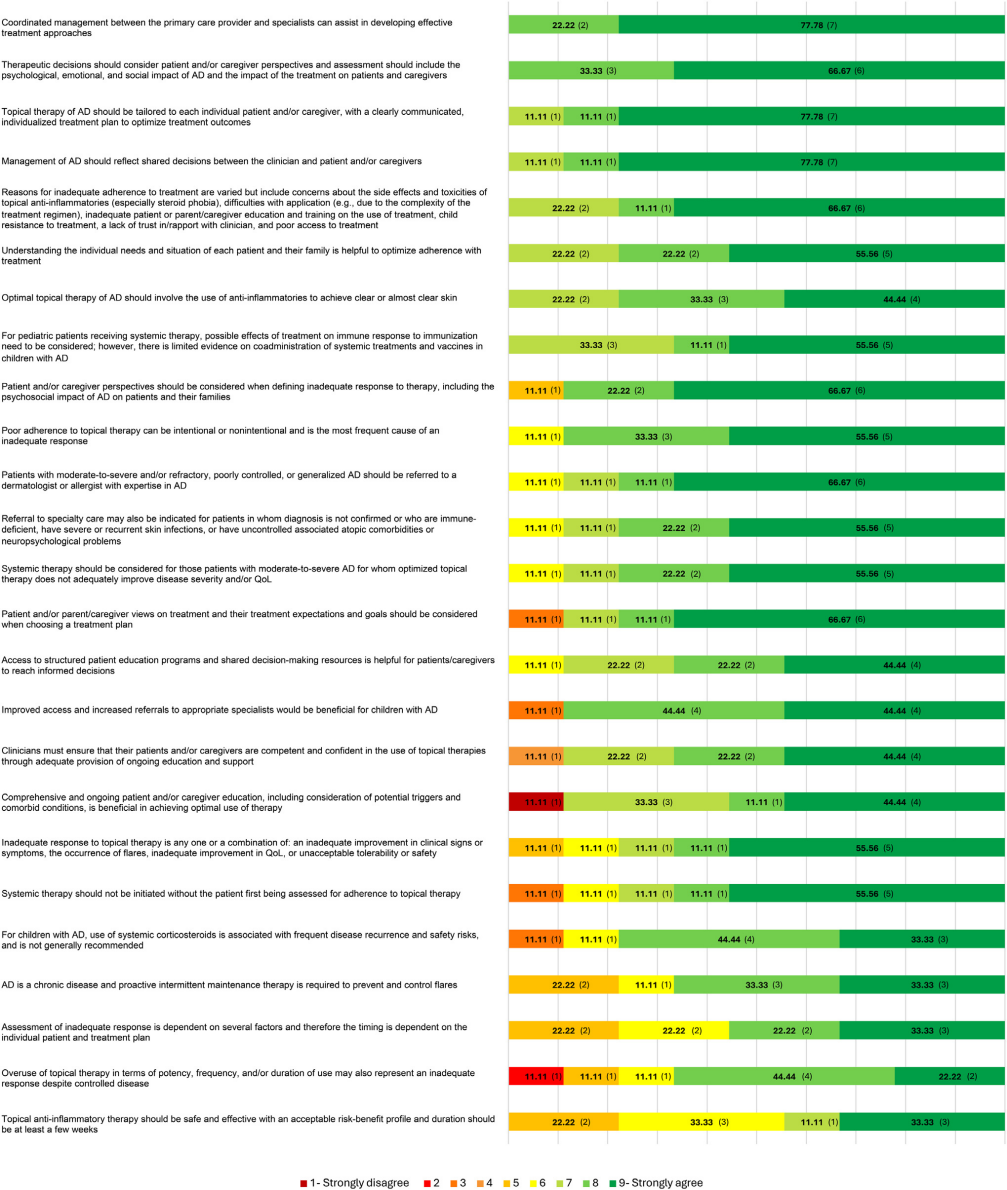
Round 1 voting results. Values in bars indicate percentage (number) of experts who allocated each score within the 9-point scale for each given statement.

**Figure 2:**
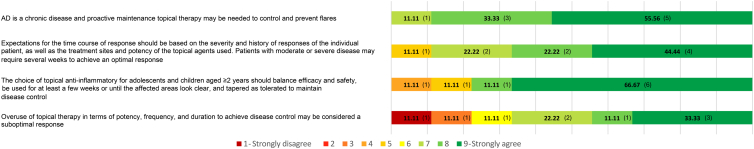
Round 2 voting results. Values in bars indicate percentage (number) of experts who allocated each score within the 9-point scale for each given statement.

After 2 rounds of voting, consensus for inclusion was reached on 24 of 25 statements (Figure 1, Table). One statement was excluded owing to a lack of consensus (at the second round of voting; Figure 2, Table). Nine statements reached the score of 7-9 by 100% of the experts. Of these, 4 were pertinent to topical therapy for the management of childhood and adolescent AD in primary care. Namely, these were the need for anti-inflammatory medication to achieve clear or almost clear skin; the need to tailor decisions about therapy to the individual patient or family; the importance of coordinated management between PCPs and specialists as part of effective treatment approaches; and the importance of patient and/or caregiver engagement in shared decision-making.

**Table:** Statements reaching consensus

Statement	Level of consensus (score 7-9), % (n)	Strength of recommendations (median)	Strength of recommendations (mean)
Coordinated management between the PCP and specialists can assist in developing effective treatment approaches.	100 (9)	9	8.78
Therapeutic decisions should consider patient and/or caregiver perspectives and assessment should include the psychological, emotional, and social impact of AD and the impact of the treatment on patients and caregivers.	100 (9)	9	8.67
Topical therapy of AD should be tailored to each individual patient and/or caregiver, with a clearly communicated, individualized treatment plan to optimize treatment outcomes.	100 (9)	9	8.67
Management of AD should reflect shared decisions between the clinician and patient and/or caregivers.	100 (9)	9	8.67
Reasons for inadequate adherence to treatment are varied but include concerns about the side effects and toxicities of topical anti-inflammatories (especially steroid phobia), difficulties with application (eg, due to the complexity of the treatment regimen), inadequate patient or parent/caregiver education and training on the use of treatment, child resistance to treatment, a lack of trust in/rapport with clinician, and poor access to treatment.	100 (9)	9	8.44
Understanding the individual needs and situation of each patient and their family is helpful to optimize adherence with treatment.	100 (9)	9	8.33
Optimal topical therapy of AD should involve the use of anti-inflammatories to achieve clear or almost clear skin.	100 (9)	8	8.22
For pediatric patients receiving systemic therapy, possible effects of treatment on immune response to immunization need to be considered; however, there is limited evidence on coadministration of systemic treatments and vaccines in children with AD.	100 (9)	9	8.22
Patient and/or caregiver perspectives should be considered when defining inadequate response to therapy, including the psychosocial impact of AD on patients and their families.	88.9 (8)	9	8.33
Poor adherence to topical therapy can be intentional or nonintentional and is the most frequent cause of an inadequate response.	88.9 (8)	9	8.33
Patients with moderate-to-severe and/or refractory, poorly controlled, or generalized AD should be referred to a dermatologist or allergist with expertise in AD.	88.9 (8)	9	8.33
Referral to specialty care may also be indicated for patients in whom diagnosis is not confirmed or who are immune-deficient, have severe or recurrent skin infections, or have uncontrolled associated atopic comorbidities or neuropsychological problems.	88.9 (8)	9	8.22
Systemic therapy should be considered for those patients with moderate-to-severe AD for whom optimized topical therapy does not adequately improve disease severity and/or QoL.	88.9 (8)	9	8.22
Patient and/or parent/caregiver views on treatment and their treatment expectations and goals should be considered when choosing a treatment plan.	88.9 (8)	9	8
Access to structured patient education programs and shared decision-making resources is helpful for patients and caregivers to reach informed decisions.	88.9 (8)	8	8
Improved access and increased referrals to appropriate specialists would be beneficial for children with AD.	88.9 (8)	8	7.89
Clinicians must ensure that their patients and/or caregivers are competent and confident in the use of topical therapies through adequate provision of ongoing education and support.	88.9 (8)	8	7.78
Comprehensive and ongoing patient and/or caregiver education, including consideration of potential triggers and comorbid conditions, is beneficial in achieving optimal use of therapy.	88.9 (8)	8	7.33
Inadequate response to topical therapy is any one or a combination of: an inadequate improvement in clinical signs or symptoms, the occurrence of flares, inadequate improvement in QoL, or unacceptable tolerability or safety.	77.8 (7)	9	7.89
Systemic therapy should not be initiated without the patient first being assessed for adherence to topical therapy.	77.8 (7)	9	7.78
For children with AD, use of systemic corticosteroids is associated with frequent disease recurrence and safety risks, and is not generally recommended.	77.8 (7)	8	7.67
∗AD is a chronic disease and proactive maintenance topical therapy may be needed to control and prevent flares.	100 (9)	9	8.44
∗Expectations for the time course of response should be based on the severity and history of responses of the individual patient, as well as the treatment sites and potency of the topical agents used. Patients with moderate or severe disease may require several weeks to achieve an optimal response.	88.9 (8)	8	7.89
∗The choice of topical anti-inflammatory for adolescents and children aged ≥2 years should balance efficacy and safety, be used for at least a few weeks or until the affected areas look clear, and tapered as tolerated to maintain disease control.	77.8 (7)	9	7.89

All 9 experts participated in both rounds of voting, providing a score of 1-9 for each statement to indicate their level of agreement (where 1 = strong disagreement and 9 = strong agreement).

∗ Statements required a second round to achieve consensus.

## Discussion

Optimizing the management of AD in pediatric patients is critical to reduce signs of inflammation, alleviate pruritus and sleep disturbance, minimize the development and/or impact of comorbidities, and improve the patient and parent/caregiver's QoL.^13^ Improved skin symptom severity, such as reduced itch or improved sleep with resultant decreased daytime drowsiness, are considered positive outcomes.^13^, ^14^, ^15^

Optimal topical therapies are the mainstay of treatment and typically sufficient for mild-moderate AD but are also part of the regimen in patients with moderate-to-severe disease. Topical corticosteroids (TCSs) are used commonly as first-line therapy in addition to nonpharmacological, basic skincare measures (eg, use of moisturizers, bathing practices and avoidance of skin irritants).^13^^,^^16^, ^17^, ^18^ Important factors to consider when choosing a TCS are patient age, AD severity, lesion location, and response to and tolerability of prior therapy, the latter of which also includes tolerability to and patient's acceptance of the vehicle formulation.^19^^,^^20^ Although some dermatologists recommend initiating treatment with the lowest potency agent, rapid, more aggressive management of flares with more potent agents may reestablish control more quickly and result in better long-term control overall.

Topical calcineurin inhibitors (tacrolimus and pimecrolimus [TCIs]) have been approved by the Food and Drug Administration (FDA) for >20 years for children ≥2 years of age and, in regions outside the US, are now also approved for use in infants ≥3 months of age.^21^^,^^22^ They are used generally for maintenance as steroid-sparing agents and for sensitive areas, such as the face (including the periorbital region) and groin.^21^ In addition, TCIs have an excellent safety profile with no evidence of increased cancer risk, despite the theoretical risk that originally led to a boxed warning.^23^^,^^24^ Other topical agents include the topical phosphodiesterase 4 inhibitors, crisaborole (FDA-approved for those ≥3 months of age) and roflumilast (currently under investigation for children ≥2 years of age), and the topical Janus kinase inhibitors, ruxolitinib (FDA-approved for those ≥12 years of age with trials ongoing for younger children) and delgocitinib (approved in Japan for children >2 years, with ongoing pediatric trials in Europe).^22^^,^^25^, ^26^, ^27^, ^28^, ^29^, ^30^, ^31^

The choice of topical anti-inflammatory agent for children and adolescents should balance efficacy and safety, be used for at least a few weeks or until the affected areas look clear, and be tapered as tolerated to maintain disease control. More potent TCS formulations in younger children may be used safely for ≤2 weeks, but the course must be monitored and limited to decrease the risk of adverse events.^19^^,^^20^ Topical nonsteroidal treatments may also be appropriate, particularly to maintain control.^19^^,^^20^

After initiation of optimal topical therapy, lesion improvement typically begins within 1 week, but continued improvement and control may take several weeks, depending on baseline severity. Treatment should be continued until at least a few days after the lesion is clear or almost clear. If a lesion is not improving within a few weeks of appropriate treatment application, treatment should be revisited, with patients and caregivers reminded about optimal frequency, quantity and application of topical therapy, general skincare, and the relapsing-remitting nature of AD.^20^

AD is a chronic disease and proactive maintenance with topical therapy may be needed to control and prevent flares. In general, the use of topical anti-inflammatory medication is reserved for lesional skin^20^; although normal-appearing, nonlesional skin in AD may still harbor barrier and immune-related abnormalities. Given their demonstrated safety to date, up to twice-daily application of nonsteroidal topical agents, including tacrolimus, pimecrolimus, or crisaborole, may be considered as maintenance treatment if tolerated.^13^ Proactive application of medium-strength TCS or tacrolimus ointment 2-3 times weekly to recurrently active areas of dermatitis once clear or almost clear may be advisable to achieve prolonged disease control.^32^, ^33^, ^34^, ^35^, ^36^ This approach may be ideal for patients who flare shortly after stopping TCS therapy. The frequency of intermittent topical therapy should be determined on an individual basis, based on clinical assessment of disease control, and in accordance with the medication label.^20^^,^^37^ In Europe, proactive therapy is an on-label indication for children (eg, with tacrolimus ointment) but is off-label in the US.^38^, ^39^, ^40^, ^41^ Proactive maintenance therapy with TCS or TCI may reduce the number of flares and increase the interval between flares in patients with moderate-to-severe AD, with the risk of side effects considered low.^32^ Although earlier studies involved fluticasone 0.05% cream or tacrolimus ointment in children, other mid-strength TCS have since been substituted with good control.^42^, ^43^, ^44^ In 2021, ruxolitinib gained FDA approval for patients aged ≥12 years with mild-to-moderate AD and currently available data support as needed use for ≤52 weeks.^13^^,^^45^ However, further evaluation is needed for proactive use and use in children <12 years of age.^45^^,^^46^ Evidence supports the use of once-daily crisaborole as maintenance treatment for mild to moderate AD in patients aged ≥3 months for ≤52 weeks, but further evaluation for its use as proactive treatment is needed.^47^ Further treatment tapering may be considered when disease severity and QoL are sufficiently improved.^48^

Patient and/or parental/caregiver input is important in achieving good treatment adherence and evaluating treatment response,^49^^,^^50^ which may include assessments of sleep quality and QoL.^51^, ^52^, ^53^ Support from a child/adolescent psychologist, if available, may also be beneficial.^54^ It should be recognized that treatment itself can affect QoL, as topical regimens may require multiple medications to address different affected areas, be complex and time consuming, and require clinician knowledge to dispense appropriate quantities for adequate treatment and to manage hypesthesia and tactile aversion.^48^^,^^55^

Tailored topical therapy for each individual patient should account for individual patient response to different treatments and specific patient/caregiver concerns.^37^^,^^56^ Treatment plans should be individualized and communicated clearly to patients and caregivers.^57^ Healthcare providers should provide a written and/or online action plan that is accessible to involved healthcare providers, patients, and caregivers.^58^^,^^59^

Comprehensive and ongoing patient and/or caregiver education, including addressing potential triggers of disease exacerbation and comorbid conditions, was acknowledged as having a key role in achieving optimal treatment. Understanding the chronic, relapsing nature of AD will help to inform the need for ongoing therapy to achieve and maintain control. Education should include guidance on the mitigation or avoidance of possible triggers, such as gentle skincare and the avoidance of soaps or detergents likely to cause irritation or contact allergy, the proper application and quantities of topical treatments (particularly with respect to TCS use), and the importance of treatment adherence.^20^^,^^37^

Expectations for the time course of response should be based on the severity of the disease and history of responses of the individual patient, as well as the treatment sites and potency of the topical agents used. Patients with moderate or severe disease may require several weeks to achieve an optimal response. Time to, and degree of, therapeutic response is dependent on several factors, including AD chronicity, severity, history of prior response, choice and potency of topical therapy, and adherence.^37^^,^^60^ Factors that negatively influence adequate response after several weeks of therapy include medication nonadherence (the most common reason), secondary infection, alternative diagnoses, or environment factors. Other reasons for inadequate response may include failure to provide a written treatment plan, the clinician dispensing an inadequate amount of treatment for the affected area of the body, inadequate medication potency, or, in rare cases, allergic contact dermatitis to the vehicle base or steroid molecule.^59^^,^^61^, ^62^, ^63^

Inadequate response to topical therapy has been difficult to define, but was deemed by consensus to include having persistent clinical signs and symptoms, frequent flares, inadequate improvement in QoL, and/or unacceptable tolerability or safety.^60^

Patient and/or caregiver perspectives should be considered when defining inadequate response to therapy, including their assessment of treatment satisfaction.^64^ While clinicians may focus on symptoms and the physical findings, patients and caregivers may have different perspectives and focus more on the pervading psychosocial burden on the family, for example, sleep disruption, school performance, social activities, and emotional well-being.^7^, ^8^, ^9^, ^10^ It is important that clinicians, patients, and families have a shared definition of treatment success.^65^

Poor adherence to topical therapy can be intentional or unintentional and is the most frequent cause of an inadequate response. Adequate adherence to treatment is a key factor in achieving a positive outcome.^66^ However, adherence to topical treatments for AD is often poor, with patients tending to overestimate their adherence.^67^ Adherence to topical therapy tends to be greatest at the start of treatment and immediately before physician visits.^68^ Adherence tends to decline with extended duration between clinic visits (approximately 30% at 8 weeks),^67^ suggesting that a follow-up visit within 1-2 months after initiating therapy is optimal for adherence.

Reasons for inadequate adherence to treatment are varied, but include concerns about the side effects and toxicities of topical anti-inflammatories (especially steroid phobia), difficulties with application (eg, due to the complexity of the treatment regimen), inadequate patient or parent/caregiver education and training on the use of treatment, child resistance to treatment, a lack of trust in or rapport with the clinician, and poor access to treatment. It is important for PCPs to educate patients and caregivers about the amount of topical medication to use per application and over time, and to prescribe an appropriate quantity to support adequate use and follow-up. The fingertip-unit, or FTU (approximately 0.5 g of cream or ointment squeezed from a standard tube with a 5-mm diameter nozzle and applied from the tip of an adult index finger to the first crease in the finger), is a simple but practical way of calculating the correct quantity of treatment to be prescribed and guiding patients and caregivers on appropriate application.^69^^,^^70^ Although somewhat inaccurate, medication dispensing history can be used to monitor adherence. Prescription medications not covered adequately by insurance may be cost prohibitive.^18^^,^^71^, ^72^, ^73^ Other mechanisms to support adherence include group educational programs, earlier follow-up visits, regular updates to written eczema action plans, and text message reminders.^37^^,^^66^

Clinicians must ensure that their patients and/or caregivers are competent and confident in the use of topical therapies through ongoing education and support. Detailed written instructions or an action plan, facilitated by the electronic medical record, provide a record of the treatment plan, which can be helpful as a decision aid. The written stepwise action plan is best reviewed at each visit and can be accomplished by support personnel if the clinician is busy.^57^^,^^66^^,^^74^ In addition to the action plan, apps targeted at teenaged patients with AD (similar to those used to improve adherence to asthma medication), videos demonstrating application of topical therapies or wraps, and various AD treatment platforms are available to answer patient/parent/caregiver questions between visits, including the National Eczema Association and Global Parents for Eczema Research.^75^, ^76^, ^77^, ^78^, ^79^ More advanced psychological tools, such as positive reinforcement and anecdotes, may help to improve treatment adherence among patients with AD.^80^ Formal education may also include age-appropriate, structured multidisciplinary educational group training programs.^81^ However, there are too few studies to support a role for self-directed educational programs. Further research is needed on treatment nonadherence in AD and how to improve it.

Understanding the individual needs and situation of each patient and their caregiver(s) is helpful to optimize adherence with treatment.^82^ Healthcare providers should explain how emollients and TCSs work, demonstrate treatment application, and help families to develop skincare routines to optimize adherence.^82^ They should also discuss lifestyle issues with their patients and their families that could preclude adherence to the regimen. For example, the parent who works at night may only participate in a morning or midday skincare routine. As another example, application of an ointment or greasy emollient in the morning may be untenable because of messiness on clothes, but a cream formulation could be substituted. Finally, some children with AD cannot tolerate the feel of an ointment, but may comply with the application of an oil and/or emollient cream.^83^ Due to variability in insurance coverage, families may face additional hurdles in initiating prescribed medication, for reasons of refusal (not on formulary) or cost after copay.^83^^,^^84^ Encouraging the family to discuss these issues with the provider allows for treatment and adherence support between appointments.

Systemic therapy should be considered for those patients with moderate-to-severe AD for whom optimized topical therapy does not adequately improve disease severity and/or QoL.^85^ Approximately one-third of pediatric patients have moderate-to-severe AD, which may not be managed adequately with topical therapy and requires the initiation of systemic therapy.^85^, ^86^, ^87^ When making the decision to begin systemic therapy, it is important to ascertain whether failure of topical treatment is due to insufficient efficacy of topical therapy (ie, disease severity) or suboptimal treatment adherence. Reasons for topical treatment failure may include insufficient access, acceptance or comprehension, time requirement, and need for excessive amounts to control disease.^18^^,^^60^^,^^83^^,^^84^

Other factors to consider before the initiation of systemic therapy include impact on QoL, associated atopic and nonatopic morbidities, risks vs benefits of such therapies, and limited access to, or intolerance of, topical medications.^88^ Alternative or concomitant diagnoses, such as contact dermatitis, infection, scabies, or an allergic disorder (eg, concomitant urticaria or multimorbid allergic rhinitis or asthma), should be considered before advancing to systemic therapy.^13^^,^^88^

Phototherapy is a form of full-body treatment that is not immunosuppressive and should also be considered for moderate-to-severe disease. Phototherapy can be challenging to incorporate into the treatment plan and the schedules of children and caregivers when therapy in a clinical setting is required 2 or 3 times weekly when treatment starts.^61^^,^^88^^,^^89^ Nevertheless, home units may be obtainable for subsequent use and home-based phototherapy reduces the need for hospital visits.^88^^,^^90^ Specifically, home-based narrowband UVB phototherapy could potentially provide a cost-effective alternative to systemic therapy, but evidence is needed to support this strategy.^90^

Systemic therapy should not be initiated without the patient first being assessed for adherence to topical therapy. If adequate adherence to topical therapy cannot be achieved and maintained despite appropriate education and support, a decision to start systemic therapy may be justified based on the clinician's understanding of the reasons for continued nonadherence and whether those reasons can justify the risk and expense of systemic therapy for a particular individual.^13^^,^^88^

The use of systemic corticosteroids for AD in children is generally not recommended, given the high safety risk and likelihood of rebound with discontinuation.^91^, ^92^, ^93^ Long-term use of systemic corticosteroids for children with severe AD is associated with adrenal suppression, negative effects on growth, hyperglycemia, inappropriate weight gain and striae, and potential effects on bone health.^91^^,^^92^ Although strongly discouraged, systemic corticosteroids may be used for brief periods for severe acute flares by practitioners with expertise in AD as a bridge to another systemic drug after appropriate discussion of any potential concerns.^91^^,^^93^

Traditional immunosuppressive drugs, such as cyclosporine, methotrexate, azathioprine, and mycophenolate mofetil, may be efficacious, but are associated with increased risk for myelosuppression, lipid abnormalities, hypertension, and/or liver or kidney toxicity; additionally, the need for serial venipuncture may have tolerability implications.^88^^,^^89^ Biologics (interleukin-4 and -13 inhibitors) and small molecule inhibitors (oral Janus kinase inhibitors) that specifically target cytokines and pathways implicated in AD pathogenesis^94^ are now available for pediatric patients with AD. These newer agents may offer improved efficacy compared with traditional therapies. Laboratory monitoring is still required for small molecule inhibitors, but not for currently approved biologics.^94^

Patient and/or parent/caregiver views on treatment and their treatment expectations and goals should be considered when choosing a treatment plan. Patients and caregivers should be informed of the therapeutic options and reasons for recommending a particular therapeutic approach so that they can be actively involved in treatment selection. Discussions and treatment planning should consider patient and caregiver goals, as well as preferences, comorbidities, and socioeconomic factors, including access.^18^^,^^95^ For example, the ability of young patients to tolerate serial blood sampling and/or injections should be considered. Parents may also have concerns regarding treatment risks and side effects, depending on the patient's overall health and other medical history.^95^^,^^96^ Ensuring that patients and caregivers have a firm understanding of their planned treatment strategy can foster proactive, collaborative, and patient-centered disease management.^50^^,^^60^^,^^97^^,^^98^

For pediatric patients receiving systemic therapy, possible effects of treatment on immune response to immunization need to be considered, especially for children aged <2 years, who have a busy immunization schedule.^99^ However, there is a paucity of studies on the coadministration of systemic treatments and vaccines in children with AD. Data from AD systemic treatments in other pediatric diseases or in adults with AD can be used as supporting evidence regarding their effect on immunization. Studies of immunosuppressant systemic therapies in other diseases have reported an effect on antibody titers in adults after immunization.^100^, ^101^, ^102^ However, data from adults with moderate-to-severe AD suggest that monoclonal antibodies targeting type 2 immunity do not affect the immune response to non-live vaccination and, in some cases, may increase the antiviral immunity vaccines provide by enhancing the T helper 1 cell response.^103^

Live-inactivated vaccines are usually contraindicated because of the theoretical risk of infection with the attenuated pathogen.^104^ International expert groups recommend that vaccines be administered before starting treatment whenever possible, with a longer time given between vaccine administration and the initiation of treatment, in the case of live vaccines, compared with inactivated vaccines.^105^ However, given that current and emerging biologics for AD target the type 2 immune pathway, which is not thought to be involved in vaccine-related immunity, and in view of the risks associated with not being immunized,^104^^,^^106^ administering live vaccines while on these biologics may be considered on a case-by-case basis after a thorough risk-benefit analysis.^105^^,^^106^ Additional studies are needed to determine whether systemic treatments affect the immune response to live attenuated vaccines in children with AD.

Universal agreement was achieved on the need for referral to a dermatologist or allergist with expertise in AD for patients with moderate-to-severe and/or refractory, poorly controlled AD.^107^, ^108^, ^109^ These children often require systemic medication for optimal management. Initial care is usually provided by PCPs, although many children are first seen in urgent care centers or even emergency rooms.^3^^,^^18^ Referral to specialty care may also be indicated for patients for whom a diagnosis is not confirmed or who are immunodeficient, have severe or recurrent skin infections, or have uncontrolled associated atopic comorbidities or neuropsychological problems.^110^

The differential diagnoses of AD include allergic contact dermatitis, seborrheic dermatitis, psoriasis, and overlapping skin disease.^111^ Incognito secondary bacterial, fungal, and/or viral skin infection can also complicate successful treatment.^112^, ^113^, ^114^ In children who are not growing well or who have a history of recurrent extracutaneous infection, primary immune or nutritional deficiencies or genodermatoses should be considered.^108^^,^^115^ Subspecialty referral is indicated in complex cases.^108^

The majority of patients with AD are not seen by an AD specialist, and this is especially the case for those without commercial insurance.^18^ Disparities in access to specialty care may exacerbate unmet treatment needs in pediatric AD. Our expert panel noted that improved access and increased referrals to appropriate specialists would be beneficial for children with AD. Pediatric dermatologists are most experienced in the evaluation and treatment of children with AD, but access may be challenging as there are a limited number of pediatric dermatologists in the US. Treatment approaches vary across physician specialties; specialists are more likely to prescribe higher-potency topicals and/or systemic therapies, regardless of patient age.^116^ Although PCPs commonly prescribe TCSs, low-potency agents are often used even when stronger medication is needed based on severity, particularly in children aged <2 years.^117^ Nonsedating antihistamines for AD generally are not helpful for pruritus,^116^ although appropriately used for concomitant allergies. Sedating antihistamines are used for their soporific effects and also do not reduce itch primarily, despite their common use.

Coordinated management between the PCP and specialists can assist in developing effective treatment approaches. Optimal outcomes are achieved when PCPs manage acute infection, work with specialists to administer and/or monitor systemic therapies, and provide immunizations through coordinated primary and secondary care.^12^

## Conclusions

The consensus of the group was that the physical signs and symptoms of AD, associated comorbidities, and overall psychosocial consequences constitute a substantial burden for affected children and their caregivers. PCPs play a vital role in the care of children and adolescents with AD by educating patients and caregivers about the chronic, relapsing nature of the disease, triggers, approaches to skincare (including appropriate use of topical medications), monitoring for complications and comorbidities, and prognosis. Appropriate, coordinated management between primary care and AD specialists is crucial to help optimize care plans, improve treatment outcomes, and alleviate the burden of AD on patients and families. The recommendations within this consensus should provide guidance for PCPs when managing pediatric patients with AD across the spectrum of disease severity.

## CRediT authorship contribution statement

**Mark Boguniewicz:** Writing – review & editing, Methodology, Conceptualization. **Moise L. Levy:** Writing – review & editing, Methodology, Conceptualization. **Lawrence F. Eichenfield:** Writing – review & editing, Methodology, Conceptualization. **Christine T. Lauren:** Writing – review & editing, Methodology, Conceptualization. **Donald Y.M. Leung:** Writing – review & editing, Methodology, Conceptualization. **Lynda C. Schneider:** Writing – review & editing, Methodology, Conceptualization. **Elaine C. Siegfried:** Writing – review & editing, Methodology, Conceptualization. **Wynnis L. Tom:** Writing – review & editing, Methodology, Conceptualization. **Amy S. Paller:** Writing – review & editing, Methodology, Conceptualization.

## Declaration of Competing Interest

M.B. reports grants from Regeneron, Sanofi, and Incyte and has participated as a consultant or advisor for AbbVie, Amgen, Dermavant, Eli Lilly and Company, Incyte, Janssen, LEO Pharma, Pfizer, Regeneron, and Sanofi Genzyme.

M.L. reports advisory board involvement and consultancy for Cassiopea, Regeneron, and UCB, was an investigator for Fibrocell, Galderma, Janssen, and Pfizer; was on a DSMB for Novan; and was a section editor and author for UpToDate.

L.E. reports research grants from AbbVie, Arcutis, Dermavant, Galderma, LEO, and Pfizer; personal/consulting fees from AbbVie, Almirall, Arcutis, Aslan, Dermavant, Forte, Galderma, Incyte, LEO Pharma, Eli Lilly and Company, Pfizer, Regeneron, and Sanofi; and has been on the board of directors and owns stocks with Forte.

C.L. has no conflicts of interest to disclose.

D.L. reports consulting with Aslan Pharmaceuticals, Evommune, Genentech, LEO Pharma, Regeneron Pharmaceuticals, Inc.; has been a principal investigator in clinical trials for Incyte; and holds a research grant with Sanofi Genzyme.

L.S. reports grants from Pfizer; participation on advisory boards for AbbVie, Amagma Therapeutics, DAIT/NIAID, LEO Pharma, and Sanofi; and has been an investigator on a clinical trial for Regeneron.

E.S. reports consulting with AbbVie, Boehringer Ingelheim, Incyte, LEO Pharma, Novan, Novartis, Pierre Fabre, Pfizer, Regeneron, Sanofi Genzyme, UCB, and Verrica; has received honoraria from Regeneron, Sanofi Genzyme, and Verrica; has served on data safety monitoring boards for LEO Pharma, Novan, Pfizer, and UCB; participated in contracted research for AI Therapeutics; served as principal investigator for Janssen; and her institution has received fees related to clinical trials from Janssen, Lilly, Pierre Fabre, Regeneron, and Verrica and in support of a 2020-2021 pediatric dermatology fellowship from Pfizer.

W.T. reports being an investigator for Regeneron, AbbVie, Janssen, Dermira, and Lilly, and a Data Safety Committee Member for LEO Pharma.

A.P. reports being an investigator or consultant for AbbVie, Boehringer Ingelheim, Bristol Myers Squibb, Catawba, Dermavant, Eli Lilly, Galderma, Janssen, LEO Pharma, Novartis, Regeneron, Sanofi/Genzyme, Seanergy, and UCB, and has served on the AbbVie and Galderma Data Safety Monitoring Boards.

The consensus development was funded by Sanofi and Regeneron. Sanofi and Regeneron had no role in the development of the consensus recommendations; collection, management, analysis, and interpretation of the literature; preparation, review, or approval of the manuscript. The authors received no financial compensation for the development and publication of this article. Medical writing support was provided by Lucid Group and funded by Sanofi and Regeneron.
