# Supplementary material for: Managing Childhood and Adolescent Atopic Dermatitis in Primary Care: A US Expert Group Consensus
Source: J Pediatr Clin Pract. 2024 Jul 10;14:200121. doi: 10.1016/j.jpedcp.2024.200121 (PMC11824657; doi:10.1016/j.jpedcp.2024.200121)
Supplement: Supplementary Appendix 1 [file mmc1.docx]

**Supplementary Appendix 1**

**A. Systematic Literature Review**

The expert panel selected key questions related to four key themes concerning the management of AD in children and adolescents in a primary care setting: defining control; current and emerging treatments; referral care pathways; and patient-caregiver experience.
A systematic literature review was conducted using Medline and Embase to identify publications that could be used to address the key questions under each theme. Search strings were defined based on terms aligned to the key questions; and relevant filters, including age of 0–12 years and application of search terms to abstract or title, were applied. Full details of search strings and applied filters for each of the key questions are provided in the following tables. The initial search results were then filtered manually to exclude publications that were irrelevant to the key questions being asked, first by review of the title and then by abstract. Results of each step, including initial search results, those filtered by title/abstract, and selected publications, were recorded in Excel format. The results of the literature review were then reviewed by the expert panel and selected publications used to inform management recommendation statements based on the initial key questions.

**B. Key Questions and Search Parameters**

**Defining Control**

Q1: How do we define treatment success/failure for short- and long-term control in pediatric patients at different ages from an investigator/healthcare provider’s perspective? ​

Q1a: How can clinical, behavioral and social effects be included? ​

Q1b: How does the patient/parent perspective influence definitions of treatment success/failure? ​

Q1c: What influence do age, skin tone, socioeconomic status and tele/remote consultations have on the success or availability of treatment?​

Q1d: What is the minimum time required on a therapy before assessing disease control?

| **Sources** | **Description of step** | **Filters applied** | **Total no. of hits** |
| --- | --- | --- | --- |
| PubMed, Embase, 2021 abstracts from AAAAI, AAD, AAP, ACAAI, ESPD, SID | Search string: (treatment OR therapy OR medication OR prescription) AND (control OR management OR success OR outcome OR effectiveness OR benefit OR success OR control OR remission OR fail OR efficacy OR flare) AND (atopic dermatitis OR AD OR eczema OR atopic eczema) AND (characterize OR define OR determine)​ | Age group: 0–12 years | 1943 |
|  |  | Search terms applied to abstract or title (with the exception of (atopic dermatitis OR AD OR eczema OR atopic eczema) which was title only) |  |
|  |  | Non-English |  |
|  |  | Review articles |  |
|  |  | Case reports |  |
|  |  | Atopic dermatitis or eczema or atopic eczema in the title |  |
|  |  | Date range: Last 10 years |  |
| PubMed, Embase, 2021 abstracts from AAAAI, AAD, AAP, ACAAI, ESPD | Search string: (quality of life OR QOL OR sleep OR ‘sleep disturbance’ OR comorbid) AND (atopic dermatitis OR AD OR eczema OR atopic eczema) ​ | Age group: 0–12 years |  |
|  |  | Search terms applied to abstract  or title |  |
|  |  | Non-English |  |
|  |  | Review articles |  |
|  |  | Case reports |  |
|  |  | Search terms applied to abstract or title (with the exception of (atopic dermatitis OR AD OR eczema OR atopic eczema) which was title only) |  |
|  |  | Date range: Last 10 years |  |
| PubMed, Embase, 2021 abstracts from AAAAI, AAD, AAP, ACAAI, ESPD | Search string: (patient reported outcomes OR parent OR proxy OR caregiver OR guardian OR add OR adherence OR compliance OR self) AND (atopic dermatitis OR AD OR eczema OR atopic eczema) ​ | Age group: 0–12 years |  |
|  |  | Search terms applied to abstract  or title |  |
|  |  | Non-English |  |
|  |  | Review articles |  |
|  |  | Case reports |  |
|  |  | Search terms applied to abstract or title (with the exception of (atopic dermatitis OR AD OR eczema OR atopic eczema) which was title only) |  |
|  |  | Date range: Last 10 years |  |
| PubMed, Embase, 2021 abstracts from AAAAI, AAD, AAP, ACAAI, ESPD | Search string: (socio OR socioeconomic OR ethnicity OR skin type OR color/colour OR 'indigenous' OR 'Native’ OR 'cultural’ OR 'sociocultural') OR (access OR disparities) OR (telemedicine OR telehealth OR tech OR teledermatology OR e-consult OR econsult) AND (atopic dermatitis OR AD OR eczema OR atopic eczema) ​ | Age group: 0–12 years |  |
|  |  | Search terms applied to abstract  or title |  |
|  |  | Non-English |  |
|  |  | Review articles |  |
|  |  | Case reports |  |
|  |  | Search terms applied to abstract or title (with the exception of (atopic dermatitis OR AD OR eczema OR atopic eczema) which was title only) |  |
|  |  | Date range: Last 10 years |  |
| PubMed, Embase, 2021 abstracts from AAAAI, AAD, AAP, ACAAI, ESPD | Search string: (Follow-up OR ‘when to measure’) AND (atopic dermatitis OR AD OR eczema OR atopic eczema) ​ | Age group: 0–12 years |  |
|  |  | Search terms applied to abstract  or title |  |
|  |  | Non-English |  |
|  |  | Review articles |  |
|  |  | Case reports |  |
|  |  | Search terms applied to abstract or title (with the exception of (atopic dermatitis OR AD OR eczema OR atopic eczema) which was title only) |  |
|  |  | Date range: Last 10 years |  |
| PubMed, Embase, 2021 abstracts from AAAAI, AAD, AAP, ACAAI, ESPD | Search string: (perception OR perspective) AND (atopic dermatitis OR AD OR eczema OR atopic eczema) | Age group: 0–12 years |  |
|  |  | Search terms applied to abstract  or title |  |
|  |  | Non-English |  |
|  |  | Review articles |  |
|  |  | Case reports |  |
|  |  | Search terms applied to abstract or title (with the exception of (atopic dermatitis OR AD OR eczema OR atopic eczema) which was title only) |  |
|  |  | Date range: Last 10 years |  |
|  | Manual filter of results based on title, abstract, additional information included | Exclusions include those not defining control | 103 |

Q2: What tools should be used to assess pediatric AD (eg, POEM or AD control test)?​

Q2a: How do tools used in clinical trials differ from tools used in clinical practice? ​

| **Sources** | **Description of step** | **Filters applied** | **Total no. of hits** |
| --- | --- | --- | --- |
| PubMed, Embase, 2021 abstracts from AAAAI, AAD, AAP, ACAAI, ESPD, Clinicaltrials.gov | Search string: (tool OR instrument OR index OR score OR measure OR survey OR measurement property/ies OR assess OR investigate OR control OR outcome) AND (atopic dermatitis OR AD OR atopic eczema OR eczema) AND (clinical trial OR trial) ​ | Age group: 0–12 years | 1346 |
|  |  | Search terms applied to abstract or title |  |
|  |  | Non-English |  |
|  |  | Review articles |  |
|  |  | Case reports |  |
| PubMed, Embase, 2021 abstracts from AAAAI, AAD, AAP, ACAAI, ESPD, Clinicaltrials.gov | Search string: (tool OR instrument OR index OR score OR measure OR survey OR measurement property/ies OR assess OR investigate OR control OR outcome) AND (atopic dermatitis OR AD OR atopic eczema OR eczema) AND (practice OR clinic OR clinical)​ | Age group: 0-12 years |  |
|  |  | Search terms applied to abstract or title |  |
|  |  | Non-English |  |
|  |  | Review articles |  |
|  |  | Case reports |  |
|  |  | Atopic dermatitis or eczema or atopic eczema in the title |  |
|  |  | Date range: Last 10 years |  |
|  | Manual filter of results based  on title, abstract, additional information included | Exclusions include those not related to tools or defining control | 79 |

**Current and Emerging Treatments**

Q1:  What defines optimal skin care and topical therapy?

Q1a: What should be the frequency and duration of treatment with topical steroids and topical calcineurin inhibitors and are these impacted by potency?

Q1b: What is the effect of (systemic) treatments on comorbidities?

| **String 1** | | | |
| --- | --- | --- | --- |
| **Source** | **Description of step** | **Filters applied** | **Total no. of hits** |
| PubMed, Embase | Search string: (skin OR epidermis OR derma OR cutaneous) AND (care OR treatment OR therapy OR management) AND (define OR characterize OR determine) AND (atopic dermatitis OR AD OR eczema OR atopic eczema) | Age group: 0–12 years | 252 |
|  |  | Search terms applied to abstract  or title |  |
|  |  | English |  |
|  |  | No case reports |  |
|  |  |  |  |
| PubMed, Embase | Manual filter of results based on title, abstract, additional information included | Exclusions include those not related to the treatments specified in the slide. Clinical trials were retained when the search was conducted to evaluate treatment regimens | 32 |

| **String 2** | | | |
| --- | --- | --- | --- |
| **Source** | **Description of step** | **Filters applied** | **Total no. of hits** |
| PubMed, Embase | Search string: (treatment OR therapy OR management) AND (comorbidity OR concomitant condition OR concomitant disease OR underlying condition OR underlying disease OR concurrent condition OR concurrent disease) AND (atopic dermatitis OR AD OR eczema OR atopic eczema) | Age group: 0–12 years | 64 |
|  |  | Search terms applied to abstract  or title |  |
|  |  | English |  |
|  |  | No case reports |  |
|  |  |  |  |
| PubMed, Embase | Manual filter of results based on title, abstract, additional information included | No results relevant to question 2 but one result identified as being relevant to question 1 | 1 |

Q2: What is the rationale for earlier initiation of systemic treatment?

Q2a: What is the potential for disease-modifying effect?

Q2b: How do comorbidities influence the choice to start a systemic treatment?

Q2c: What are the risks of developing comorbidities as a result of treatment?

| **Source** | **Description of step** | **Filters applied** | **Total no. of hits** |
| --- | --- | --- | --- |
| PubMed, Embase, AAAAI, ESPD, ACAAI | Search string: (systemic treatment OR systemic therapy OR injection treatment OR injection therapy OR infusion treatment OR infusion therapy OR oral treatment OR oral therapy OR antibody treatment OR antibody therapy) AND (atopic dermatitis OR AD OR eczema OR atopic eczema) | Age group: 0–12 years | 93 |
|  |  | Search terms applied to abstract  or title |  |
|  |  | English |  |
|  |  | No case reports |  |
|  |  |  |  |
| PubMed, Embase, AAAAI, ESPD, ACAAI | Manual filter of results based on title, abstract, additional information included | Exclusions include those not related to systemic treatment and not AD/eczema | 35 |

Q3: What are the potential effects of age on treatment efficacy?

Q3a: What differences are there between age groups (birth to <2y, 2y to <6y, 6y to <12y) regarding treatment efficacy, safety or chosen treatment?

Q3b: How does efficacy and safety relate to potential developmental differences in the immune system?

Q3c: How does efficacy and safety relate to potential differences in social behavior between age groups?

Q3d: What are the potential adverse events/impacts of treatment on neurological development, growth and the immune system?

| **Source** | **Description of step** | **Filters applied** | **Total no. of hits** |
| --- | --- | --- | --- |
| PubMed, Embase | Search string: age AND (treatment OR therapy OR management) AND (efficacy OR effectiveness OR success) AND (safety OR adverse effects OR side effects) AND (atopic dermatitis OR AD OR eczema OR atopic eczema) | Age group: 0–12 years | 150 |
|  |  | Search terms applied to abstract  or title |  |
|  |  | Non-English |  |
|  |  | Case reports |  |
|  |  |  |  |
| PubMed, Embase | Manual filter of results based on title, abstract, additional information included | Exclusions include those not related to AD and those not covering the correct age groups | 54 |

Q4: What factors need to be considered regarding vaccinations in children receiving systemic treatments?

Q4a: How do systemic treatments impact the immune response to live or inactivated viral vaccines, or risks associated with a live vaccine?

Q4b: Should decisions on coadministration of treatment and vaccines be data-driven?

| **Source** | **Description of step** | **Filters applied** | **Total no. of hits** |
| --- | --- | --- | --- |
| PubMed, Embase | Search string: (vaccine OR vaccination) AND (systemic treatment OR systemic therapy OR injection treatment OR injection therapy OR infusion treatment OR infusion therapy OR oral treatment OR oral therapy OR antibody treatment OR antibody therapy) | Age group: 0–12 years | 44 |
|  |  | Search terms applied to abstract  or title |  |
|  |  | English |  |
|  |  | No case reports |  |
|  |  |  |  |
|  | Manual filter of results based on title, abstract, additional information included |  | 2 |

**Referral Care Pathways**

Q1: What is a typical pediatric AD patient journey (including presentation, diagnosis, management and/or referral)?

Q1a: What are the criteria that lead to escalation of care?

Q1b: How is escalation of care influenced by disease severity, age, time since diagnosis, patient/caregiver input, and quality and understanding of treatment regimen?

| **Sources** | **Description of step** | **Filters applied** | **Total no.  of hits** |
| --- | --- | --- | --- |
| PubMed, Embase, 2021 abstracts from AAAAI, AAD, AAP, ACAAI, ESPD, SID, ICER, Expert Insights | Search string: (patient journey OR clinical journey OR patient pathway OR care pathway OR clinical presentation OR diagnosis OR management OR referral) AND (atopic dermatitis OR AD OR eczema OR atopic eczema) | Age group: 0–12 years | 644 |
|  |  | Search terms applied to abstract  or title |  |
|  |  | Non-English |  |
|  |  | Review articles |  |
|  |  | Case reports |  |
|  |  | Atopic dermatitis or eczema or atopic eczema in the title |  |
|  |  | Date range: Last 10 years |  |
|  | Manual filter of results based on title, abstract, additional information included | Exclusions include those not related to referral/care pathways | 45 |

Q2: What are the roles of multidisciplinary team members (including dermatologists/ pediatric dermatologists, allergist-immunologists, contact dermatitis specialists, behavioral health clinicians, nurse practitioners/physician assistants/registered nurses, sleep specialists, rehab specialists, dieticians) in difficult-to-manage AD?

Q2a: What role does an allergy evaluation (IgE-mediated allergies and allergic contact dermatitis) have and when should allergy be evaluated?

| **Sources** | **Description of step** | **Filters applied** | **Total no.  of hits** |
| --- | --- | --- | --- |
| PubMed, Embase, 2021 abstracts from AAAAI, AAD, AAP, ACAAI, ESPD, SID, ICER, Expert Insights | Search string: (multi disciplinary OR multidisciplinary OR allergist OR dermatologist OR immunologist OR dermatitis specialist OR contact dermatitis specialist OR behavioral health clinician OR nurse) AND (atopic dermatitis OR AD OR eczema OR atopic eczema) | Age group: 0–12 years | 902 |
|  |  | Search terms applied to abstract or title |  |
|  |  | Non-English |  |
|  |  | Review articles |  |
|  |  | Case reports |  |
| PubMed, Embase, 2021 abstracts from AAAAI, AAD, AAP, ACAAI, ESPD, SID, ICER, Expert Insights | Search string: (atopic dermatitis OR AD OR eczema OR atopic eczema) AND (atopic march OR allergy OR food trigger OR food allergy OR inhalant trigger) | Age group: 0–12 years |  |
|  |  | Search terms applied to abstract or title |  |
|  |  | Non-English |  |
|  |  | Review articles |  |
|  |  | Case reports |  |
|  |  | Atopic dermatitis or eczema or atopic eczema in the title |  |
|  |  | Date range: Last 10 years |  |
|  | Manual filter of results based  on title, abstract, additional information included | Exclusions include those not related to AD/allergy testing  in AD | 79 |

Q3: What are the most frequent HCP points of contact for pediatric patients with AD?

Q3a: How are HCP points of contact and management/referral impacted by age, race and socioeconomic status?

Q3b: What is the role of a primary care provider (pediatrician or GP) in AD management? Does this depend on the practitioner’s confidence/education regarding different therapies?

| **Sources** | **Description of step** | **Filters applied** | **Total no. of hits** |
| --- | --- | --- | --- |
| PubMed, Embase, 2021 abstracts from AAAAI, AAD, AAP, ACAAI, ESPD, SID, ICER, Expert Insights | Search string: (atopic dermatitis OR AD OR eczema OR atopic eczema) AND (referral OR care OR management) AND (healthcare provider OR primary care) AND (pediatrician OR general practitioner OR dermatologist OR pediatric dermatologist OR allergist OR nurse) | Age group: 0–12 years | 34 |
|  |  | Search terms applied to abstract or title |  |
|  |  | Non-English |  |
|  |  | Review articles |  |
|  |  | Case reports |  |
|  | Manual filter of results based  on title, abstract, additional information included | Exclusions include those not related to referral/care pathway/HCP contacts | 12 |

**Patient-Caregiver Experience**

Q1: How is the quality of life of pediatric AD patients and their families (including siblings) influenced separately by treatment/escalation of care, suboptimal disease control and the disease itself (at different ages)?

Q1a: What are the effects on parent/caregiver sleep, and how can itching and sleep issues be managed?

Q1b: How does skincare place a burden on families?

| **Sources** | **Description of step** | **Filters applied** | **Total no. of hits** |  |
| --- | --- | --- | --- | --- |
| PubMed, Embase, Expert insights | PubMed search string: (quality of life OR QoL OR sleep) AND (family OR caregiver OR sibling OR proxy OR guardian OR patient) AND (atopic dermatitis OR AD OR eczema OR atopic eczema) | Age group: 0–12 years | 405 |  |
|  |  | Search terms applied to abstract  or title |  |  |
|  |  | Non-English |  |  |
|  |  | Case reports |  |  |
|  |  |  |  |  |
|  | Embase search string: (skin OR epidermis OR derma OR cutaneous) AND (care OR treatment OR therapy OR management) AND (define OR characterize OR determine) AND (atopic dermatitis OR AD OR eczema OR atopic eczema) |  |  |  |
|  | Manual filter of results based on title, abstract, additional information included | Exclusions include those not related to AD, eczema or QOL | 105 |  |

Q2: How does parent disease education and awareness of disease-modifying therapies influence management of pediatric AD?

Q2a: What are the best practices for sharing objective assessments with parents?

Q2b: What is the importance of managing parents’ concerns (eg, food allergies, environmental triggers,) and expectations regarding treatment, disease control, and adverse events and the role of shared decision-making?

Q2c: What aspects of AD should parents be educated about (eg, pathophysiology, disease natural history, itch-scratch cycle, relapses, comorbidities, prognosis)?

Q2d: How do patient/parent satisfaction and patient/parent phobias towards a prescribed regimen affect adherence and what is the role of the doctor-patient relationship (education and management discussions at initial diagnosis)?

| **Sources** | **Description of step** | **Filters applied** | **Total no. of hits** |  |
| --- | --- | --- | --- | --- |
| PubMed, Embase, Expert Insights | PubMed search string 1: (quality of life OR QoL OR sleep) AND (family OR caregiver OR sibling OR proxy OR guardian OR patient) AND (atopic dermatitis OR AD OR eczema OR atopic eczema) | Age group: 0–12 years | 166 |  |
|  |  | Search terms applied to abstract  or title |  |  |
|  |  | Non-English |  |  |
|  |  | Case reports |  |  |
|  |  |  |  |  |
|  | PubMed search string 2: (parent OR caregiver OR family OR proxy OR guardian) AND (education OR knowledge OR awareness OR phobia OR fear OR concern OR psychology) AND (management OR therapy OR treatment) AND (atopic dermatitis OR AD OR eczema OR atopic eczema)​ |  |  |  |
|  |  |  |  |  |
|  | Embase search string 1:  (parent OR caregiver OR family OR proxy OR guardian) AND (education OR knowledge OR awareness OR phobia OR fear OR concern OR psychology) AND (management OR therapy OR treatment) AND (atopic dermatitis OR AD OR eczema OR atopic eczema) |  |  |  |
|  | Embase search string 2: (parent OR caregiver OR family OR proxy OR guardian) AND (clinician OR doctor OR healthcare practitioner OR specialist) AND (relationship OR interaction OR partnership OR decision making) AND (atopic dermatitis OR AD OR eczema OR atopic eczema) |  |  |  |
|  | Manual filter of results based on title, abstract, additional information included | Exclusions include those not related to AD, or eczema or education | 55 |  |

Q3: How do disease triggers and comorbidities (atopic and non-atopic) change with age?

Q3a: How do triggers impact atopic (asthma, food allergy, and allergic rhinitis) and non-atopic AD comorbidities?

| **Sources** | **Description of step** | **Filters applied** | **Total no. of hits** |  |
| --- | --- | --- | --- | --- |
| PubMed, Embase, Expert Insights | Search string: (trigger OR triggering factor OR exacerbating factor OR aggravating factor) OR (comorbidity OR concomitant condition OR concomitant disease OR underlying condition OR underlying disease OR concurrent condition OR concurrent disease) AND (atopic dermatitis OR AD OR eczema OR atopic eczema) | Age group: 0–12 years | 324 |  |
|  |  | Search terms applied to abstract or title |  |  |
|  |  | Non-English |  |  |
|  |  | Case reports |  |  |
|  |  |  |  |  |
|  | Manual filter of results based on title, abstract, additional information included | Exclusions include those not related to AD or eczema or triggers | 43 |  |
